# Mortality rates in people with intellectual disabilities

**DOI:** 10.23889/ijpds.v1i1.333

**Published:** 2017-04-19

**Authors:** Rachael Williams, Jessie Oyinlola, Pauline Heslop, Gyles Glover

**Affiliations:** 1 Clinical Practice Research Datalink; 2 Norah Fry Research Centre, University of Bristol; 3 Learning Disabilities Observatory Team, Public Health England

## Objectives

A growing body of evidence highlights a disparity in mortality rates for people with intellectual disability (ID) compared with the general population. However, national data for England is lacking. The objective of this study was to provide evidence on mortality rates in people with ID.

## Approach

Patients registered for at least a day during 01/04/10-31/03/14 at a GP practice contributing to the Clinical Practice Research Datalink (CPRD) and consenting to linkage were included. Patients with ID were identified via Read codes. Date and cause of death were identified using linked Office of National Statistics mortality data. Crude mortality rates, life expectancy and indirectly age/sex standardised mortality ratios (SMR) were calculated with 95% confidence intervals (CI), overall, by ICD10 chapter, for frequently occurring causes, and those classified as avoidable.

## Results

11 million person-years were included (0.5% for patients with ID) and 98,035 deaths occurred (0.7% in patients with ID). The mortality rate for patients with ID was 11.2 per 1,000 population, 1.3 times the rate for those without ID, with an associated SMR of 3.2 (95% CI 2.93.4). Life expectancy was 65.5 years (95% CI 61.969.2)for patients with ID and 85.3 years for those without (95% CI 85.285.4). Mortality rates were higher in patients with ID in all age/sex groups, with larger differences for younger ages. Patients with ID had higher cause-specific mortality rates across all ICD10 chapters, with highest SMRs for congenital malformations (72.9, 95% CI 55.194.7), nervous system diseases (9.8, 95% CI 7.812.1) and mental disorders (5.4, 95% CI 3.97.3). Circulatory deaths were the most frequent, with ischaemic heart disease (SMR 2.2, 95% CI 1.62.8) and cerebrovascular disease (SMR 3.3, 95% CI 2.34.5) most prominent. A higher proportion of deaths were classified as avoidable for patients with ID (44.7%, 95% CI 41.048.5%) compared to those without (21.0%, 95% CI 20.721.3). 

## Conclusion

National English data confirm that patients with ID have higher mortality rates than those without. Mortality rates for patients with ID were higher across all age/sex groups and causes, with almost half of deaths classified as avoidable.

